# Urinary metabolomic analysis to identify preterm neonates exposed to histological chorioamnionitis: A pilot study

**DOI:** 10.1371/journal.pone.0189120

**Published:** 2017-12-06

**Authors:** Claudia Fattuoni, Carlo Pietrasanta, Lorenza Pugni, Andrea Ronchi, Francesco Palmas, Luigi Barberini, Angelica Dessì, Roberta Pintus, Vassilios Fanos, Antonio Noto, Fabio Mosca

**Affiliations:** 1 Department of Chemical and Geological Sciences, University of Cagliari, Cagliari, Italy; 2 NICU, Department of Clinical Sciences and Community Health, Fondazione IRCCS Ca’ Granda Ospedale Maggiore Policlinico, Università degli Studi di Milano, Milan, Italy; 3 Department of Medical Sciences and Public Health, University of Cagliari, Cagliari, Italy; 4 Maternal-Neonatal Department, Neonatal Intensive Care Unit, Puericulture Institute and Neonatal Section, AOUCA University Hospital of Cagliari, Cagliari, Italy; Hopital Robert Debre, FRANCE

## Abstract

**Objective:**

Chorioamnionitis is a leading cause of preterm birth worldwide, with higher incidence at lower gestational ages. An early and reliable diagnosis of histological chorioamnionitis (HCA) in preterm infants may be helpful in guiding postnatal management, especially the administration of prophylactic antibiotics to prevent early-onset sepsis. The main aim of this study was to investigate metabolomic analysis of urines collected in the first 24 hours of life as diagnostic tool of HCA.

**Methods:**

Gestational age-, birth weight-, delivery mode- and sex- matched (1:2) preterm neonates (< 35 weeks’ gestation) born to mothers with or without HCA were enrolled from an observational study. Gas chromatography-mass spectrometry (GC-MS)-based metabolomic analysis was performed on urine samples non-invasively collected in the first 24 hours of life. Univariate analysis, partial least square discriminant analysis (PLS-DA) and its associated variable importance in projection (VIP) score were performed. The most affected metabolic pathways were examined by Metabolite Sets Enrichment Analysis (MSEA).

**Results:**

Fifteen cases (mean GA 30.2 ± 3.8 weeks, mean BW 1415 ± 471.9 grams) and 30 controls (mean GA 30.2 ± 2.9 weeks, mean BW 1426 ± 569.8 grams) were enrolled. Following univariate analysis, 29 metabolites had a significantly different concentration between cases and controls. The supervised PLS-DA model confirmed a separation between the two groups. Only gluconic acid, an oxidation product of glucose, was higher in cases than in controls. All other VIP metabolites were more abundant in the control group. Glutamate metabolism, mitochondrial electron transport chain, citric acid cycle, galactose metabolism, and fructose and mannose degradation metabolism were the most significantly altered pathways (*P* < 0.01).

**Conclusions:**

For the first time, urinary metabolomics was able to discriminate neonates born to mothers with and without HCA. The identification of specifically altered metabolic pathways may be helpful in understanding metabolic derangement following chorioamnionitis.

## Introduction

Chorioamnionitis is a leading cause of preterm birth worldwide, with higher incidence at lower gestational ages [1]. The term chorioamnionitis is used to refer to an intrauterine infection/inflammation (IUI) occurring between the maternal tissues and the fetal membranes (choriodecidual space) or in the fetal annexes (chorioamniotic membranes, amniotic fluid, umbilical cord) [2]. Affecting up to 50–70% of live births at 24–27 weeks of gestation, chorioamnionitis contributes significantly to neonatal morbidity and mortality. Many neonatal adverse outcomes, such as death, early- and late-onset sepsis (EOS and LOS), bronchopulmonary dysplasia, necrotizing enterocolitis (NEC) and cerebral palsy, have been differently associated with chorioamnionitis [3–7].

Histological examination of the placenta constitutes the gold standard for diagnosis, however the results are available only several days after birth [8]. Prenatal diagnosis of IUI using clinical criteria (i.e. the diagnosis of clinical chorioamnionitis) provides insufficient specificity and sensitivity with any combination of maternal or fetal clinical signs [9,10]. To overcome this potentially high rate of incorrect clinical diagnoses, different tools have been recently proposed, such as the combination of clinical and/or laboratory signs into predictive clinical scores, an extended use of amniocentesis and the introduction of new biochemical markers [11–13].

Certainly, an early, reliable diagnosis of IUI, before or immediately after birth, could improve the individualized care of the neonates. This appears particularly true for what concerns the management of postnatal antibiotic treatment for prevention of EOS. Currently, international guidelines recommend diagnostic evaluation, including blood culture, and administration of empiric antibiotic therapy for all term and preterm neonates born to mothers with clinically suspected chorioamnionitis to avoid possible occurrence of EOS, whose incidence is very high in the presence of chorioamnionitis [14,15]. Given the well-known difficulty in diagnosing chorioamnionitis based on clinical signs, a situation of over-treatment of otherwise healthy neonates with antibiotics in the first 2–3 days of life is not sporadic [16]. Considering the increasing development of multidrug-resistant pathogens and the growing evidences that link early exposure to antibiotics with adverse short- and long-term outcomes, such as toxicity (e.g. aminoglycosides effects on kidneys and ear), intestinal dysbiosis (ultimately possibly leading to NEC) and a possible increased incidence of atopy or wheezing, a more reliable, fast and early diagnosis of chorioamnionitis would be necessary [17–20].

In the last few years, a metabolomic approach was used by some authors to identify metabolic changes associated with early spontaneous preterm birth and to better understand the pathophysiological mechanism of chorioamnionitis [21,22]. In 2010, Romero et al. [23] reported that the presence of intra-amniotic infection/inflammation was associated with an altered amniotic fluid metabolite composition. More recently, in a study by Dudzik et al. [24], amniotic fluid metabolomic analysis identified women with and without chorioamnionitis.

In the present study, gas chromatography-mass spectrometry (GC-MS)-based metabolomic analysis was performed on urine samples non-invasively collected in the first 24 hours of life from preterm neonates exposed or not exposed to histological chorioamnionitis (HCA), with a dual intent: first, to investigate the ability of urinary metabolomics to discriminate between neonates born to mothers affected by HCA and neonates born to mothers without HCA within the first 24 hours of life; second, to analyze neonatal urinary metabolic products altered by HCA, in order to understand the influence of chorioamnionitis on newborn’s metabolism.

## Materials and methods

### Study population and design

This was a pilot, nested case-control study in the context of a single-center observational prospective cohort study, performed to evaluate outcomes in neonates exposed to HCA. The observational study was conducted at the Neonatal Intensive Care Unit (NICU) of Fondazione IRCCS Ca’ Granda Ospedale Maggiore Policlinico of Milan, Italy, from November 2011 to September 2015. The study protocol was approved by the Ethics Committee of the Hospital and a written informed consent was obtained from parents before inclusion in the study. All procedures were in accordance with the Helsinki Declaration of 1975, as revised in 2008.

All inborn neonates with a gestational age (GA) < 35 weeks and/or a birth weight (BW) ≤ 1500 g admitted to the NICU were consecutively enrolled in the observational study. Exclusion criteria were being outborn, the presence of major congenital anomalies, lack of parental consent or a missing pathological examination of fetal adnexa.

A group of neonates among those enrolled in the observational study was retrospectively selected for metabolomic analysis on the basis of histological examination of the placenta, in order to compare the metabolomic profile of urine collected from neonates born to mothers with HCA (cases) with that of a group of neonates born to mothers without HCA (controls). Cases were matched 1:2 with controls for GA at birth, BW, sex, and delivery mode. The sample size was established following the method reported by Julious et al. [25]. HCA was diagnosed and graded using Redline’s classification [8]. GA was established on the basis of best obstetric estimates, including last menstrual period and first or second trimester ultrasonography.

Clinical data were prospectively collected from the electronic medical records for the entire birth cohort, including case-subjects and controls for the present study.

Urine was collected within the first 24 h of life from all the neonates enrolled in the observational study until sample size for metabolomic analysis was obtained. Urine samples were collected using a cotton ball in the disposable diaper that was checked every 60–90 minutes for the presence of urine. Cotton balls contaminated by stools were discarded and replaced. Urines were squeezed from the cotton ball using a sterile 1 mL syringe and immediately frozen and stored at -80°C until analysis, conducted at the Department of Chemical and Geological Sciences of University of Cagliari, Italy.

### Sample preparation and analysis

Urine samples were thawed at room temperature and vortexed to homogenize. 150 μL of urine were transferred in a 2 mL Eppendorf tube with 800 μL of urease solution (1 mg/mL), vortexed for 1 min, and sonicated for 30 min. 800 μL of cold methanol was added, the mixture was vortexed for 1 min, and centrifuged at 4°C, 14 000 rpm for 10 min. 1200 μL of supernatant were successively transferred in glass vials and evaporated to dryness in an Eppendorf vacuum centrifuge. 30 μL of a 0.24 M solution of methoxylamine hydrochloride in pyridine were added to each vial, samples were vortex mixed 1 min and left to react for 17 h at room temperature. 30 μL of *N*-Methyl-*N*-trimethylsilyltrifluoroacetamide (MSTFA) were added, the mixture was vortex mixed for 1 min and then allowed to react for 1 h at room temperature. The derivatized samples were diluted with 600 μL of hexane containing tetracosane (0.01 mg/mL) as internal standard, just prior to GC-MS analysis.

Samples were analyzed using a MS Agilent 5975C interfaced to the GC 7820 equipped with a DB-5ms column (J & W); injector temperature at 230°C, detector temperature at 280°C, helium carrier gas flow rate of 1 mL/min. The GC oven temperature program was 90°C initial temperature with 1 min hold time and ramping at 10°C/min to a final temperature of 270°C with 7 min hold time. 1 μL of the derivatized sample was injected in split mode (1:20). After a solvent delay of 3 min mass spectra were acquired in full scan mode using 2.28 scans/s with a mass range of 50–700 Amu. Each acquired chromatogram was analysed using the free software AMDIS (Automated Mass Spectral Deconvolution and Identification System; http://chemdata.nist.gov/mass-spc/amdis). Successively, each peak was identified by comparing relative mass spectrum and retention time with those stored in an in-house made library including 296 metabolites. Other metabolites were identified using NIST08 (National Institute of Standards and Technology's mass spectral database), and Golm Metabolome Database (GMD; http://gmd.mpimp-golm.mpg.de/). This strategy allowed for the detection of 131 compounds: 113 accurately identified, 8 unknown compounds matching equally unknown compounds contained in GMD, and 10 unknown molecules recurring in every sample. AMDIS analysis produced an electronic sheet data matrix (Microsoft^®^ Excel^®^, Microsoft Co, Redmond Washington DC, USA) that was submitted to univariate and multivariate statistical analysis.

### Statistical analysis

Continuous clinical and demographic data were tested for normality and compared between cases and controls using *t* test (for variable maternal age) or Mann-Whitney U test (for variables BW, GA and umbilical venous blood pH value). Fisher's exact test was used for categorical variable. Data were analyzed using Excel Version 14.0 (Microsoft Corp. Redmond, WA, USA), and *P* values < 0.05 were considered as statistically significant. For the analysis of metabolomic data, the Excel data matrix containing 131 metabolites was processed using the integrated web-based platform MetaboAnalyst 3.0 [26]. Univariate analysis, partial least square discriminant analysis (PLS-DA) and its associated variable importance in projection (VIP) score were performed. To determine the optimal number of components needed to build the PLS-DA model, the sum of squares captured by the model (R^2^) and the cross-validated R^2^ (Q^2^) were determined. The most affected metabolic pathways were examined by Metabolite Sets Enrichment Analysis (MSEA) in MetaboAnalyst 3.0. MSEA uses a set of predefined metabolic pathways to identify significant changes in functionally related metabolites. Data were input in MSEA as a list of 98 metabolites identified by their Human Metabolome Database (HMDB) ID [27] and the quantitative enrichment analysis (QEA) was applied using the pathway-associated metabolite sets containing 88 metabolite sets based on normal metabolic pathways [28].

## Results

A total of 15 cases (group 1) and 30 controls (group 2) underwent metabolomic analysis. The mean GA was 30.2 ± 3.8 weeks for neonates born to mothers with HCA and 30.2 ± 2.9 weeks for controls, while the mean BW was 1415 ± 471.9 grams for cases and 1426 ± 569.8 for controls.

There was no significant difference between cases and controls in demographic characteristics, prenatal management or clinical indicators of adaptation to extra-uterine life (Table 1), despite neonates born to mothers with HCA presented a slightly, non-significant higher prevalence of intubation in delivery room. Three out of 15 (20%) neonates exposed to HCA developed an EOS and 2 (13.3%) of them developed a LOS, while no case of EOS and 5 cases of LOS (16.6%) were recorded in the control group.

**Table 1:** Clinical characteristics and neonatal outcomes of the study population. Group 1: cases, neonates born to mothers with histological chorioamnionitis; group 2: controls. Fisher’s exact test for categorical variables; *t* test or Mann-Whitney U test for continuous variables (OR: odds ratio; CI: confidence interval).

	group 1 (n = 15)	group 2 (n = 30)	*P* value	OR (95% CI)
maternal age (mean ± SD)	35.2 ± 4.1	33.2 ± 7.3	0.23	-
ethnic group				-
- caucasian	12 (80%)	24 (80%)	1	1
- south-american	0	2 (6.6%)	n.a.	n.a.
- black	1 (6.6%)	1 (3.3%)	0.61	(0.12–34.8)
- asian	2 (13.3%)	3 (10%)	0.74	1.38 (0.1–13.6)
clinical choriamnionitis	6 (40%)	6 (20%)	0.16	2.67 (0.54–12.8)
positive vaginal swab^1^	5 (33.3%)	11 (36.6%)	0.82	(0.18–3.74)
maternal antibiotics	9 (60%)	13 (43.3%)	0.29	1.96 (0.47–8.48)
pre-eclampsia	1 (6.6%)	4 (13.3%)	0.65	0.46 (0.01–5.49)
prenatal steroids	13 (86.6%)	26 (86.6%)	1	1
caesarean section	12 (80%)	28 (93.3%)	0.18	0.28 (0.02–2.92)
gestational age (weeks^2^)	30.2 ± 3.8	30.2 ± 2.9	0.97	-
birth weight (grams)	1415 ± 471.9	1426 ± 569.8	0.95	-
male gender	10 (66.6%)	20 (66.6%)	1	-
twin	5 (33.3%)	18 (60%)	0.09	0.33 (0.07–1.44)
small for gestational age	0	4 (13.3%)	n.a.	n.a.
Apgar at 1 min (median, range)	5 (1–9)	6 (1–9)	0.20	-
Apgar at 5 min (median, range)	8 (3–10)	8 (3–10)	0.14	-
umbilical venous blood pH value	7.37 ± 0.03	7.34 ± 0.06	0.19	-
resuscitation in delivery room^3^	12 (80%)	24 (80%)	1	1
oxygen in delivery room	10 (66.6%)	13 (43.3%)	0.14	2.6 (0.61–12.1)
endotracheal intubation in delivery room	7 (46.6%)	6 (20%)	0.06	3.5 (0.76–16.7)
surfactant therapy in delivery room	2 (13.3%)	4 (13.3%)	1	1
early-onset sepsis	3 (20%)	0	n.a.	n.a.
late-onset sepsis	2 (13.3%)	5 (16.6%)	0.81	0.77 (0.06–5.57)
death before discharge	3 (20%)	1 (3.3%)	0.2	7.25 (0.5–393)

^1)^ isolated pathogens (1 or more per swab): *Streptococcus agalactiae*, *Escherichia coli*, *Pseudomonas aeruginosa*, enterococci, fungi, *Ureaplasma spp*, genital *Mycoplasmas*

^2)^ completed weeks of gestation

^3)^ at least ventilation with mask

By means of GC-MS metabolomic analysis, urine samples collected during the first 24 hours of life from neonates born to mothers with HCA were compared with those of controls through univariate and multivariate analysis.

Following univariate analysis, 29 metabolites had a significantly different concentration between cases and controls. As reported in Table 2, all identified metabolites, except for gluconic acid, had a lower concentration in urine from neonates born to mothers with HCA.

**Table 2:** Urinary metabolomic profile in neonates born to mothers with histological chorioamnionitis (group 1, cases) *vs*. controls (group 2). Univariate analysis (*t* test) between the groups. *P* value with (W) is calculated by the Mann-Whitney-Wilcoxon test.

metabolite	1 *vs*. 2	*P* value
Monosaccharide E	Down	0.0001
Succinic acid	Down	0.0002
Mannose	Down	0.0012(W)
4-Hydroxyproline	Down	0.0015
Arabinose	Down	0.0017
Erythritol	Down	0.0019
Threonic acid	Down	0.0019(W)
Citric acid	Down	0.0019(W)
Arabitol	Down	0.0021
Glucoheptonic acid 1,4-lactone	Down	0.0021(W)
Malic acid	Down	0.0023
Erythronic acid	Down	0.0026
3,4-Dihydroxybutyric acid	Down	0.0032
Threitol	Down	0.0079(W)
Oxalic acid	Down	0.0081
Gluconic acid	**Up**	**0.0109(W)**
N-Acetylglucosamine	Down	0.0109(W)
Ethanolamine	Down	0.0118
Glyceric acid	Down	0.0130
3-Hydroxy-3-methylglutaric acid	Down	0.0149(W)
Xylose	Down	0.0149(W)
Lyxonic acid	Down	0.0149(W)
N-Acetylneuraminic acid	Down	0.0149(W)
Glycolic acid	Down	0.0160
Glycine	Down	0.0214
Lactic acid	Down	0.0312
U C	Down	0.0341
Glucuronic acid	Down	0.0346(W)
2-Hydroxyglutaric acid	Down	0.0486(W)

**Fig 1:**
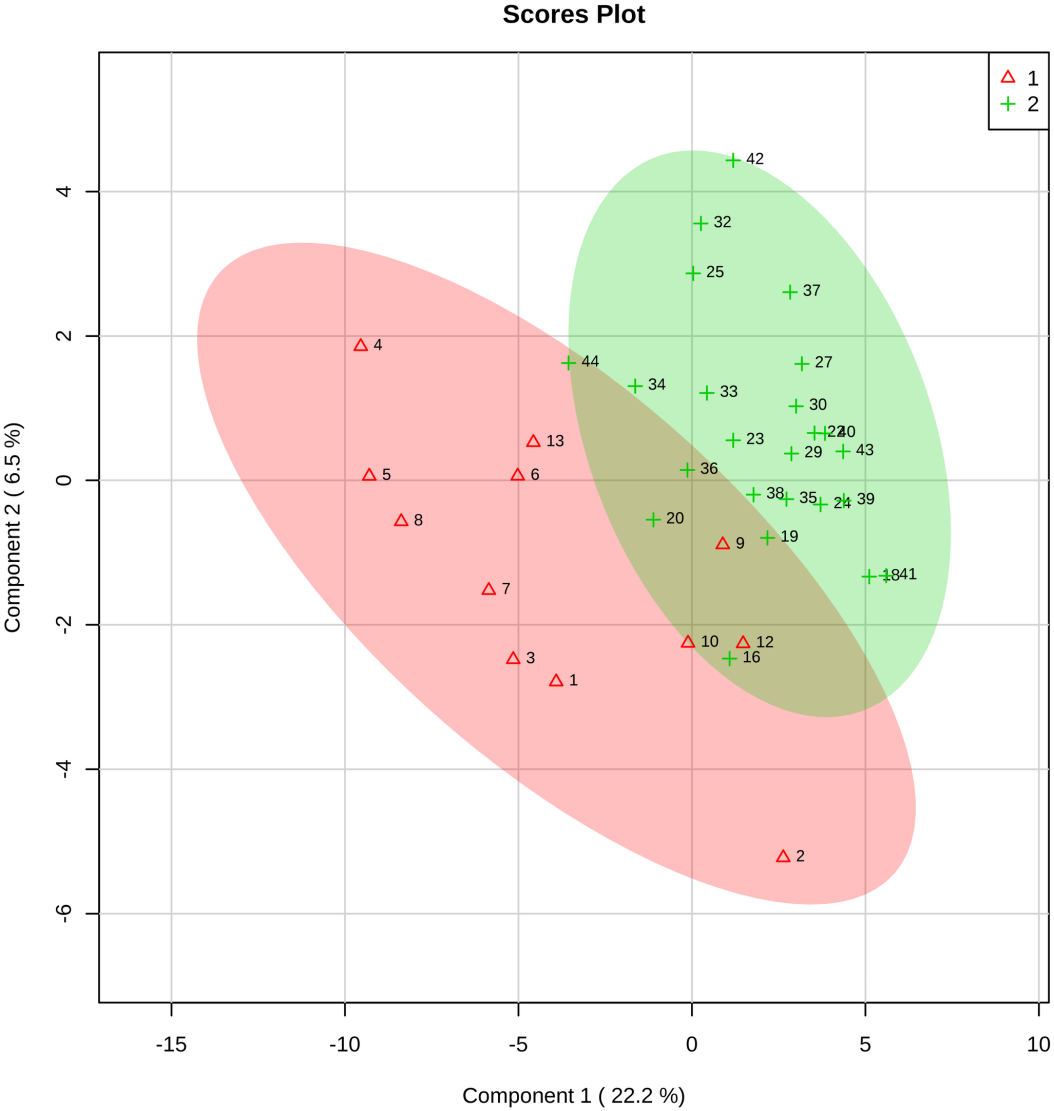
2D scores plot showing PLS-DA discrimination between urine samples of neonates born to mothers with histological chorioamnionitis (group 1, red) and urine samples of controls (group 2, green). The shaded areas indicate the 95% confidence regions.

Thirty-six samples (12 cases and 24 controls) underwent PLS-DA analysis (3 samples from group 1 and the 6 matching controls were removed from the analysis, inasmuch as 3 samples from the control group resulted as outliers). The supervised PLS-DA model showed a separation between cases and controls as reported in Fig 1.

This model was best described by the fourth principal component, showing accuracy = 0.79, R^2^ (goodness of fit) = 0.87, and Q^2^ (goodness of prediction) = 0.39. The cross validation (CV) method applied was 10-fold CV and the corresponding performance measure resulted in *P* = 0.12, probably due to the low number of samples.

**Fig 2:**
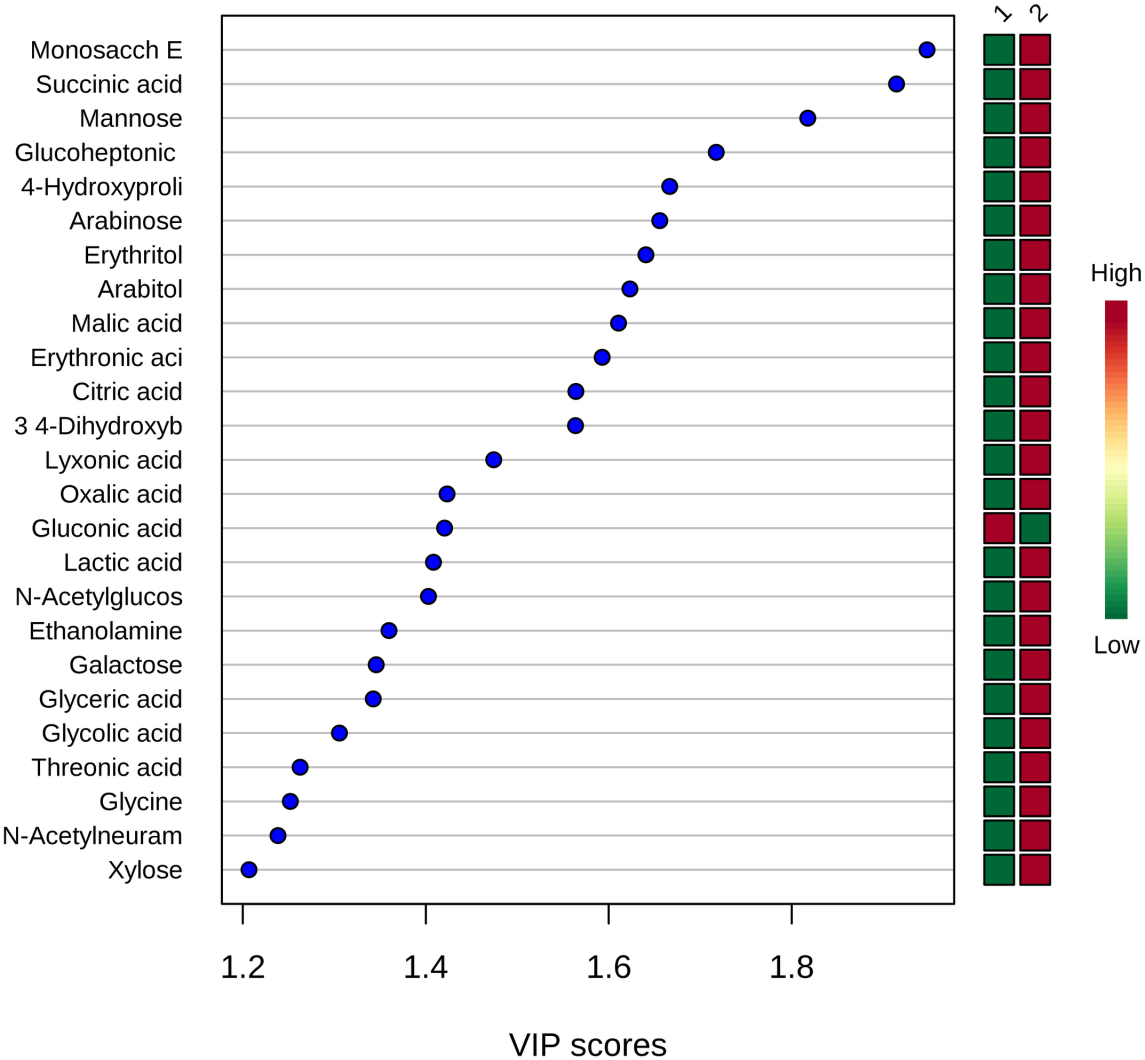
Summary plot showing the most important metabolites ranked based on the variable importance in projection (VIP) score. The mini heatmap on the right indicate their concentration variations within the groups (group 1, cases: neonates born to mothers with histological chorioamnionitis; group 2, controls).

Among the 131 detected metabolites, those most responsible for the discrimination between the two groups are reported, in order of importance, in Fig 2.

According to their chemical classification, VIP metabolites shown in Fig 2 can be grouped as carbohydrates (unknown monosaccharide E, mannose, arabinose, galactose, xylose), amino acids (4-hydroxyproline and glycine), sugar-related polyols (erythritol, arabitol), carboxylic acids (succinic, glucoheptonic, malic, erythronic, citric, 3,4-dihydroxybutyric, lyxonic, oxalic, gluconic, lactic, glyceric, glycolic, threonic, *N*-acetylneuraminic acid), and amine derivatives (*N*-acetylglucosamine, ethanolamine). Only gluconic acid, an oxidation product of glucose, was higher in cases than in controls. All other VIP metabolites were more abundant in the control group.

**Fig 3:**
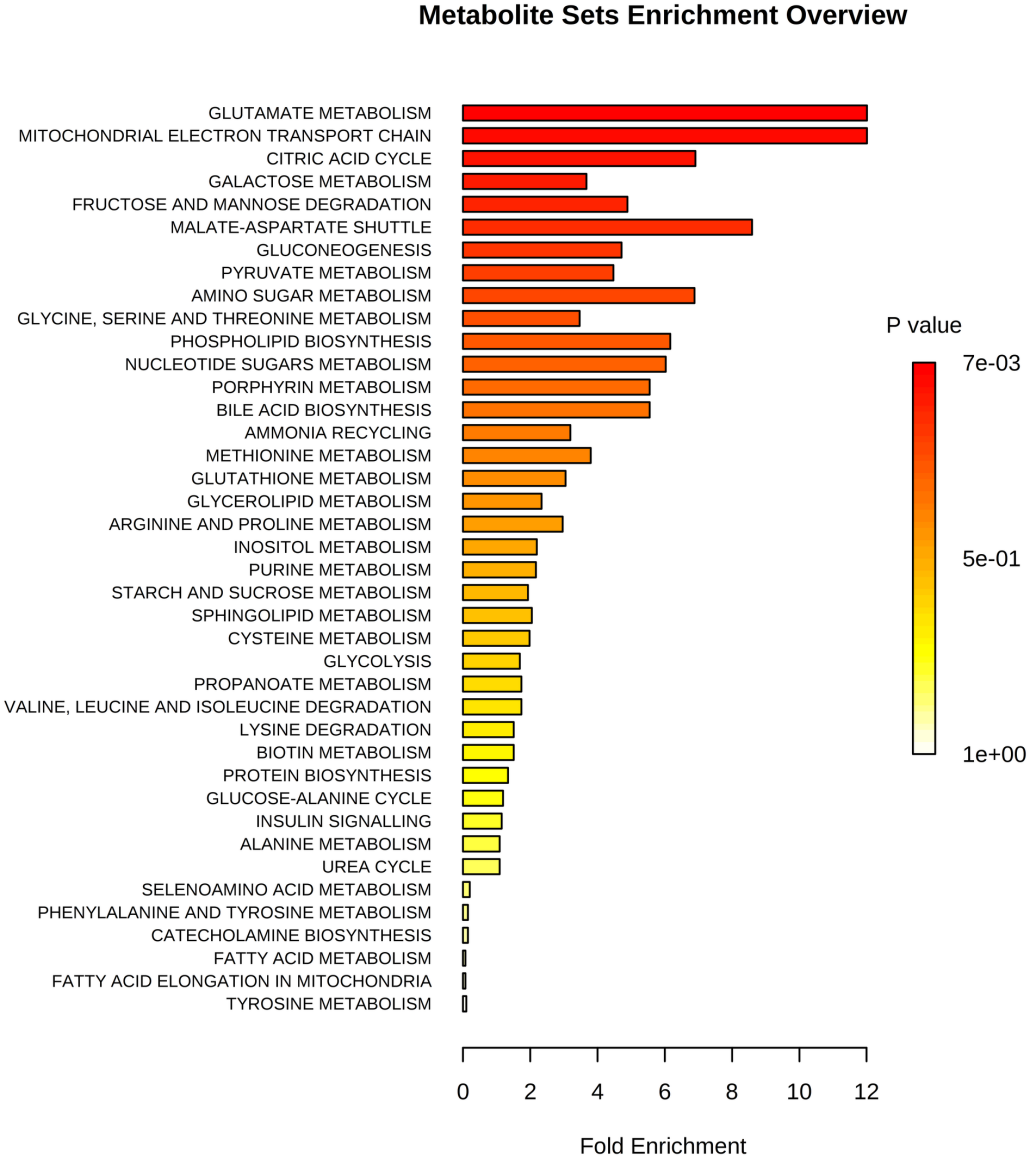
Graphical summary of Metabolite Sets Enrichment Analysis (MSEA). Altered metabolic pathways in neonates born to mothers with histological chorioamnionitis compared with controls are shown in order of decreasing *P* value.

Subsequently, as groups of metabolites are usually associated with biological functions or pathways, an enrichment analysis was performed in order to facilitate a functional interpretation. The MSEA results are shown in Fig 3. The horizontal bar graph summarizes the most significant metabolite sets identified. The bars are colored based on their *P* values and the bar length is proportional to the fold enrichment. Glutamate metabolism, mitochondrial electron transport chain, citric acid cycle (i.e. tricarboxylic acid cycle, TCA), galactose metabolism, and fructose and mannose degradation metabolism were the most significantly altered pathways (*P* < 0.01) (Table 3).

**Table 3:** Overview of the metabolic pathways most significantly altered (*P* < 0.01) in neonates born to mothers with histological chorioamnionitis compared with controls. Total indicates the total number of metabolites listed in the pathways; hits indicate the number of significant metabolites identified in the pathways; the *P* value is based on the enrichment analysis; FDR indicates false discovery rate.

metabolite set	Total	Hits	*P* value	FDR
glutamate metabolism	18	1	0.00017481	0.0034963
mitochondrial electron transport chain	15	1	0.00017481	0.0034963
citric acid cycle	23	5	0.001101	0.012996
galactose metabolism	25	7	0.0014468	0.012996
fructose and mannose degradation	18	3	0.0016245	0.012996
malate-aspartate shuttle	8	1	0.0021231	0.014154
gluconeogenesis	27	4	0.0034039	0.019451
pyruvate metabolism	20	4	0.0046021	0.02301
amino sugar metabolism	15	1	0.0067573	0.030032
glycine, serine and threonine metabolism	26	5	0.009046	0.036184

## Discussion

Chorioamnionitis is a prenatal condition that has been associated with different adverse neonatal outcomes [29]. The strongest association has been clearly established between chorioamnionitis, either clinical or histological, and a higher incidence of EOS [6,30], providing the rationale for antibiotic treatment of neonates born to mothers with clinical signs of chorioamnionitis.

Unfortunately, no reliable early marker of HCA is currently available and risk stratification of neonates is based on clinical risk factors such as GA, the presence of preterm pre-labor rupture of membranes and/or of clinical signs of chorioamnionitis in the mother [31]. This behavior may ultimately lead to a poorly specific identification of babies really born to mothers with HCA, that could distinctively benefit from a tailored support or pharmacological approach [11] while, conversely, it may expose a high number of not-at-risk neonates to unnecessary postnatal antibiotic therapy [32]. In this context, the possibility of diagnosing HCA shortly after birth with a non-invasive neonatal biomarker may greatly improve the management of both HCA-exposed and not exposed neonates.

The objectives of the present study were: 1) to establish if metabolomic analysis of urines collected non-invasively during the first 24 hours of life was able to discriminate neonates born to mothers affected by HCA, in order to target more appropriately antibiotic prophylaxis not relying on a clinical diagnosis of chorioamnionitis that often yields false positive and negative results; 2) to characterize neonatal metabolic pathways specifically altered by the presence of HCA itself. Urinary metabolomics has been recently used, even by our group, to investigate several clinical conditions in neonates, such as intrauterine growth restriction, sepsis or cytomegalovirus infection [33–35]. To the best of our knowledge, this is the first time urinary metabolomics has been applied to identify neonates exposed to HCA.

In the study population, case-control matched by GA, BW and sex, no significant difference with regard to prenatal risk factors, obstetric perinatal management, and postnatal well-being was recorded. Nonetheless, twenty per cent of babies born to mothers with HCA developed EOS, confirming the strong link between HCA and EOS already underlined by many authors.

As for the metabolomic analysis, metabolomics fingerprinting of urine was able to discriminate between neonates born to mothers with and without HCA. All identified metabolites, except for gluconic acid, had a lower concentration in urine from neonates born to mothers with HCA compared with controls, even if the statistical significance of the proposed model was affected by the low number of samples.

Moreover, the two most affected metabolic pathways were correlated to the energy metabolism, including the citric acid cycle and the mitochondrial electron transport chain. Low concentrations of succinic, citric and malic acid in urine samples from neonates born to mothers with HCA could be related to neonatal mitochondrial dysfunction, possibly due to a systemic inflammatory status triggered by microbial antigens frequently responsible for HCA and similar to that seen in septic patients [36].

Furthermore, the influence of bacterial metabolism may be hypothesized. In our study, the observed metabolites that may be associated with bacterial metabolism include *N*-acetylglucosamine, *N*-acetylneuraminic acid, 3,4-dihydroxybutyric acid, ethanolamine and arabinose, which were found decreased in pathological samples. *N*-acetylglucosamine is an amide formed by the condensation of glucosamine and acetic acid and it is a key component of cellular wall of both Gram-positive and Gram-negative bacteria. In particular, *N*-acetylglucosamine is a major component of cell wall peptidoglycan, which contains alternating residues of *N*-acetylglucosamine and *N*-acetylmuramic acid [37]. Reduced amounts of *N*-acetylglucosamine may be related to the higher demand caused by bacterial growth. In addition, the alteration observed for the fatty acid 3,4-dihydroxybutyric acid and the monosaccharide arabinose seems to be a sign of dysbiosis, as was recently shown by De Angelis et al. [38].

A lower concentration of the hexoses mannose and galactose (along with the not precisely identified monosaccharide E) in urine samples from neonates born to mothers with HCA compared with controls may be attributed to the presence of bacteria in the amniotic cavity. In similar fashion, in a study by Romero et al. [23], the metabolomic analysis of amniotic fluid from patients with preterm labor and intra-amniotic infection/inflammation showed a decreased amount of hexoses, namely mannose and galactose, that might derive from the increased demand of carbohydrates due to bacterial growth. Similar findings were reported by Dudzik et al. [24] in the LC-MS metabolomic analysis of amniotic fluid: lower amount of hexoses were detected in women with chorioamnionitis compared with healthy subjects. The authors hypothesized that low hexoses concentration could be explained by the fact that glucose, due to infection, was used as energy source by bacteria and neutrophils. The same hypothesis was advanced by Abehsera et al. [39] in a study performed in 45 pregnant women who had undergone diagnostic amniocentesis for risk of intra-amniotic infection: the authors reported that glucose concentration in amniotic fluid was inversely correlated to the severity of chorioamnionitis, because of a probably increased consumption of glucose in the infected fetus.

Moreover, our results documented a significant activation of the glycerophospholipid metabolism in neonates born to mothers with HCA. This is in agreement with the results obtained in 2016 by Prince et al. [40], who studied the metabolic pathways altered in the placental membranes of mothers affected by chorioamnionitis and that experienced a preterm delivery. The authors suggested that the increase in glycerophospholipid metabolism results in increased production of arachidonic acid, which promotes inflammation and the synthesis of prostanoids that are associated with labor.

Finally, the increase in gluconic acid in urine samples from neonates born to mothers with HCA could be related to the microbial activity and the pentose phosphate pathway [41]. Glucose is usually converted to pyruvic acid through glycolysis. Bacteria, however, can use other metabolic pathways to get there to pyruvic acid and, in particular, the pentose shunt and the Entner–Doudoroff (ED) pathway [42]. Some forced aerobic bacteria are incapable of phosphorylation of glucose to glucose-6-phosphate and use glucose through an initial oxidation to gluconic acid, which is then phosphorylated to 6- phosphogluconic and converted into pyruvic acid through the pentose shunt or via ED pathway. As of gluconic acid, a recent study on metabolomics and NEC demonstrated a remarkable increase in gluconic acid in urine samples from babies affected by NEC [43]: this may furtherly support the high importance of bacterial metabolites in the discrimination between sick and healthy subjects.

A significant strength of this study is the use of a non-invasively collected biological sample such as urine to perform highly informative metabolomic analysis in the first 24 hours of life. This strategy may allow for the investigation of the metabolic derangement induced by prenatal conditions such as IUI with minimal influence on postnatal management. Nonetheless, we are aware of some weaknesses of our study. The sample size is small, but adequate for a pilot study; further investigations in large groups of neonates are needed to confirm our results. Another limitation may be the lack of microbiological analysis on placental tissues to identify a more reliable association between intrauterine infection, HCA and a subsequent neonatal metabolic fingerprint. However, especially in preterm pregnancies, it is known that most cases of HCA are a consequence of microbial invasion of the amniotic cavity and chorioamnionic membranes [44].

To the best of our knowledge, this is the first time urinary metabolomics has been applied to identify neonates exposed to HCA. In this study, GC-MS-based metabolomic analysis of urine non-invasively collected in the first 24 hours of life from preterm neonates was able to distinguish those born to mothers with and without HCA. Furthermore, this particular approach may prove to be a powerful tool to better understand the altered metabolic pathways associated to chorioamnionitis.

## Supporting information

S1 File Pugni_raw_mass_data_matrix.xlsx. Matrix spreadsheet (Microsoft Excel^®^, Microsoft Co, Redmond, WA, USA) containing the detected metabolites and their respective concentrations. First column contains metabolites names, first row samples identifier, second row class identifier, 1 defining chorioamnionitis samples and 2 control samples. Samples in this file are completely anonymized.
(XLSX)
